# Enhanced Photocatalytic Activity of CQDs-Modified Layered g-C_3_N_4_/Flower-like ZnO Heterojunction for Efficient Degradation of Ciprofloxacin

**DOI:** 10.3390/nano15070550

**Published:** 2025-04-04

**Authors:** Qing Liu, Wei Deng, Hai Zhang, Jiajun Fang, Yushi Xie, Congwen Liu, Xiaochen Han, Xiaoling Xu, Zuowan Zhou

**Affiliations:** 1 School of Chemistry, Key Laboratory of Advanced Technologies of Materials (Ministry of Education), Southwest Jiaotong University, Chengdu 610031, China; liuqing@my.swjtu.edu.cn (Q.L.); weideng@my.swjtu.edu.cn (W.D.); zhanghai@swjtu.edu.cn (H.Z.); jjfang@my.swjtu.edu.cn (J.F.); xys0417@my.swjtu.edu.cn (Y.X.); lan129cm@my.swjtu.edu.cn (C.L.); hxc.swjtu.edu.cn@my.swjtu.edu.cn (X.H.); zwzhou@swjtu.edu.cn (Z.Z.); 2 Yibin Research Institute, Southwest Jiaotong University, Yibin 644000, China

**Keywords:** CQDs, ZnO, g-C_3_N_4_, heterojunction, photocatalysis, ciprofloxacin

## Abstract

Photocatalytic degradation has the advantages of high efficiency and stability compared with traditional antibiotic treatment. Therefore, the development of efficient and stable photocatalysts is essential for antibiotic degradation in water treatment. In this study, layered g-C_3_N_4_/flower-like ZnO heterojunction loaded with different amounts of CQDs (C_x%_CNZO (x = 1, 2, 3, 4)) were precisely synthesized at room temperature. The as-prepared photocatalyst showed enhanced performance in degrading ciprofloxacin (CIP). The heterojunction with CQDs loaded at 3 wt% (C_3%_CNZO) achieved a 91.0% removal rate of CIP at 120 min under a sunlight simulator illumination, and the photodegradation reaction data were consistent with the first-order kinetic model. In addition, cycling experiments confirmed that the C_3%_CNZO heterojunction had good reusability and photocatalytic stability after four cycles. According to the experimental results, superoxide radical (•O_2_^−^) was the main active species involved in CIP degradation. Furthermore, C_3%_CNZO was found to conform to a type II electron transfer pathway. Finally, the possible degradation pathways of CIP were analyzed. This work may provide an effective strategy for the removal of various antibiotics in water treatment.

## 1. Introduction

Antibiotics, as a type of medicine, are usually used to prevent bacterial diseases and treat bacterial infections. Therefore, Ciprofloxacin (CIP) is extensively used in the medical industry and animal husbandry due to its excellent bactericidal effect and outstanding antibacterial spectrum [1]. Usually, CIP cannot be absorbed by humans and other organisms completely. A large amount of untreated residual antibiotics flow into surface water and groundwater, and eventually return to human domestic water [2,3], causing a series of health problem and environmental issue [4,5,6]. Therefore, there is an urgent need to develop advanced and efficient technologies to treat antibiotics in water bodies. Common antibiotic treatment technologies include physical adsorption [7,8], biodegradation [9,10], chemical oxidation [11], etc. However, due to the antibacterial properties and stable chemical structure of antibiotics, it is difficult to completely decompose antibiotics by commonly used conventional methods [6]. These methods may also produce secondary contamination and super-bacteria [12,13]. Semiconductor photocatalysis technology, as a fantastic water-treatment technology, is considered a promising technology for antibiotic treatment due to its easy access to reaction conditions (light and room temperature), high redox capacity, safety, and environmental friendliness [14,15,16]. In addition, it is widely recognized for its low cost and high efficiency [17].

Common semiconductor catalysts include ZnO [18,19], TiO_2_ [20,21], BiOX (X = Cl, Br, I) [22,23], LDH [24], and others. Among the various semiconductor photocatalysts, ZnO is particularly distinguished due to its exceptional optical, electrical, catalytic [25], antimicrobial [26], and biocompatible properties. Compared with TiO_2_, which is the most widely used on the market today, ZnO has a lower production cost and higher electron mobility. The electron mobility of ZnO is at least two orders of magnitude higher than TiO_2_, which has led to a wide range of applications in the field of waste-water treatment [27,28]. However, the wide band gap of ZnO (~3.20 eV, absorbing only ultraviolet light) [29] and the fast recombination of photogenerated electron/hole (e^−^/h^+^) pairs, as well as its instability under weak acid or strong alkali conditions, have limited the practical application of pure ZnO [30]. Common modification strategies include defect engineering [31], doping [32], and heterostructure building [33,34]. Graphite carbon nitride (g-C_3_N_4_), as a novel two-dimensional layered non-metallic semiconductor polymer, offers high chemical stability and a relatively narrow band gap (~2.70 eV) [35,36]. Due to the characteristics of its energy bands, g-C_3_N_4_ is the most suitable material for coupling with ZnO [37,38,39]. The composite of ZnO and g-C_3_N_4_ not only enhances the photocurrent, but also effectively mitigates photo-corrosion [40]. As a result, the photocatalytic efficiency of the obtained heterojunction will be substantially improved compared with that of pure ZnO or g-C_3_N_4_ [41,42,43].

As a member of carbon-based materials, carbon quantum dots (CQDs) are widely used in photocatalytic systems due to their ultra-small size, up-conversion effect, and low cost [44]. Due to their electronic properties, CQDs can not only expand the photo-response range, but also separate and migrate the generated charges in the catalytic system, making them good photocatalytic auxiliary material [45]. Therefore, the use of CQDs to replace precious metals or modify semiconductors is an effective way to improve the photocatalytic activity of catalysts [46].

**Scheme 1:**
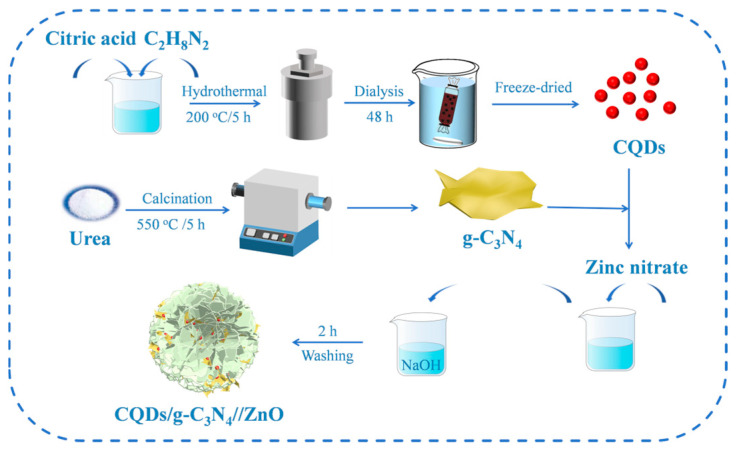
The synthesis pathway of photocatalyst material.

In this study, we chose flower-like ZnO with highly active exposed crystal surfaces as the substrate. The CQDs-modified enhanced layered g-C_3_N_4_/flower-like ZnO (C_x%_CNZO (x = 1, 2, 3, 4)) heterojunction catalysts were synthesized at room temperature to investigate their photocatalytic performance and reusability. The synthesis routes of the heterojunction are shown in Scheme 1. The results show that the incorporation of this with CQDs expands the light absorption range of the photocatalyst and increases the production of photoelectrons, which leads to the good photocatalytic and cycling properties of the materials. The heterojunction with CQDs loaded of 3 wt% (C_3%_CNZO) achieved a 91.0% removal rate of CIP at 120 min under a sunlight simulator illumination, and the photodegradation reaction data were consistent with the first-order kinetic model. Even after four repeated cycles, the catalytic performance of the catalysts could still be maintained above 80.0%. Meanwhile, the degradation pathway of CIP was deducted by LC-MS. Finally, a possible mechanism of the photocatalyst was proposed by active species capture experiments and electron spin resonance (EPR) experiments.

## 2. Materials and Methods

### 2.1. Materials

Urea (CH_4_N_2_O), citric acid (C_6_H_8_O_6_·H_2_O), ethylenediamine (C_2_H_8_N_2_), zinc nitrate (Zn (NO_3_)_2_·6H_2_O), sodium hydroxide (NaOH), isopropyl alcohol (IPA), ethylenediamine tetra-acetic acid disodium salt dihydrate (EDTA-2Na), and 1,4-benzoquinone (BQ) were purchased from Chengdu Kelong Chemicals Co., Beijing, China. (Chengdu, China). CIP was purchased from the China National Pharmaceutical Group Chemical Reagent Co. All reagents in the experiments were of analytical grade without any disposal. And the water used in the experiment was deionized water (DI).

### 2.2. Catalyst Preparation

#### 2.2.1. Preparation of CQDs

Citric acid (C_6_H_8_O_7_) was used as a carbon source; 1.05 g of citric acid was dissolved in 10 mL of deionized water, 335 μL of ethylenediamine (C_2_H_4_N_2_) was added dropwise in a fume hood, and the reaction was carried out hydrothermally at 200 °C for 5 h. The CQDs obtained from the reaction were dialyzed for 48 h, and the CQDs powder was later obtained by freeze-drying, and was prepared as a 1 mg/mL solution of CQDs.

#### 2.2.2. Preparation of g-C_3_N_4_

To prepare g-C_3_N_4_ using urea as the precursor, 5 g urea (CH_4_N_2_O) was calcined in air at 550 °C for 2 h. The heating rate in the muffle Furnace was controlled at 5 °C/min, and the temperature was then decreased to room temperature at the same rate. Finally, the resulting sample was ground into a powder.

#### 2.2.3. Preparation of CQDs Modified Layered g-C_3_N_4_/Flower-like ZnO

First, 35 mg g-C_3_N_4_ was dispersed into 150 mL of deionized water by ultrasonication for 2 h. Then, different amounts of CQDs (1 wt%, 2 wt%, 3 wt%, and 4 wt%) were added; 2.23 g of zinc nitrate was added as well and stirred in the suspension to ensure uniform dispersion. Afterwards, the suspension was rapidly poured into 150 mL of sodium hydroxide solution (dissolved 0.075 mol NaOH in this solution), kept at 25 °C, and magnetically stirred for 2 h. Next, the suspension was centrifuged and washed by deionized water (DI) and absolute ethanol. Finally, the collected samples were dried at 60 °C to yield CQDs-modified layered g-C_3_N_4_/flower-like ZnO powder, noted as C_x%_CNZO (x = 1, 2, 3, 4). Under the same conditions, pure ZnO, CQDs/ZnO, and g-C_3_N_4_/ZnO powders were prepared without adding CQDs or g-C_3_N_4_. The CQDs/ZnO and g-C_3_N_4_/ZnO powders were denoted as C_3%_ZO and CNZO, respectively.

### 2.3. Characterization

The morphology of C_3%_CNZO was characterized by a scanning microscope (SEM, JSN-6330F JEOL, Kikuchi, Japan) at 15 kV. The exposed crystalline surfaces of the heterojunctions were analyzed by transmission electron microscopy (TEM, JSM-2100F JEOL Japan) at an accelerating voltage of 200 kV. The crystal structure of the sample was tested by X-ray diffraction (XRD, D8 X-Ray, Bruker, Bremen, Germany) with Cu Kα radiation in the range of 10–80°. The chemical element composition and valence states of C_3%_CNZO were analyzed by X-ray photoelectron spectroscopy (XPS, Thermo Scientific K-Alpha, Oxford, UK), where C 1s (C−C binding energy of 284.8 eV) were used as the charge correction reference. For UV-visible diffuse reflectance (UV-Vis DRS) spectra, a UV-visible spectrometer (UV-2600, Shimadzu, Tokyo, Japan) with a BaSO_4_ reference was used.

### 2.4. Photocatalytic Degradation Experiments

The photocatalytic performance of the C_x%_CNZO (x = 1, 2, 3, 4) heterojunction photocatalyst was evaluated by using CIP as the degradation pollutant. A 300 W xenon lamp was used as the simulated light source to drive the photocatalyst for the degradation of CIP at room temperature. During the photocatalytic degradation, 50 mg catalyst was added to a 50 mL CIP (10 mg/L) solution to prepare a 1 g/L suspension. The adsorption/desorption equilibrium was reached by stirring for 30 min under dark conditions. During the degradation, 3 mL of the reaction solution was taken every 20 min, centrifuged, and 2.5 mL of the supernatant was collected. The CIP concentration at different times was measured by the UV-vis spectrophotometer at an absorption wavelength of 272.5 nm. According to the Lambert−Beer law, the absorbance is proportional to the concentration, so the degradation rate of CIP was calculated by the following equation:

$$D=\frac{\left(C_{0}-C_{t}\right)}{C_{0}}\times 100\%=\frac{\left(A_{0}-A_{t}\right)}{A_{0}}\times 100\%$$

where *D* is the degradation rate, *C*_0_ is the initial concentration of the CIP solution, *C_t_* is the solution concentration of CIP at moment t, and *A*_0_ and *A_t_* correspond to the absorbance of the CIP solution at the initial and t moments, respectively.

### 2.5. Active Species Capturing and Recycling Experiments

The main active species generated in the photocatalyst were captured by adding scavengers in the catalytic process. Usually, the active species capture experiments were similar to photocatalytic degradation experiments. We used IPA, EDTA-2Na, and BQ to capture •OH radicals, h^+^, and •O_2_^−^ radicals. The contrast experiment was taken on the same conditions without any scavenger.

We measured the recyclability and stability of C_3%_CNZO through the four cyclic degradations of CIP. After each cycle, the used C_3%_CNZO was collected by centrifugation, washed and then dried at 60 °C for use in the next cycle. After four recycles, the used C_3%_CNZO was further characterized by SEM, XRD, FTIR, and XPS and compared with fresh samples to verify their morphology and structural stability.

### 2.6. Electrochemical Testing

Transient and photocurrent density, and electrochemical impedance spectroscopy (EIS) with a frequency range of 1000–100,000 Hz were measured by an electrochemical workstation (CHI 760E, Shanghai, China). The platinum, the Ag/AgCl, and the sample under test were used as counter, reference, and working electrodes, respectively. The electrolyte was Na_2_SO_4_ solution (0.5 mol/L). The working electrode was prepared as follows: First, 10 mg of the catalyst sample was mixed with 1 mL of absolute ethanol and 100 μL of Nafion solution to form a slurry. Then, the mixture was drop-coated on a 2 cm × 2 cm fluorine-doped tin oxide (FTO) glass electrode. Finally, the prepared electrode was dried at 60 °C to acquire the working electrode.

## 3. Results

### 3.1. Characteristics of Materials

**Figure 1:**
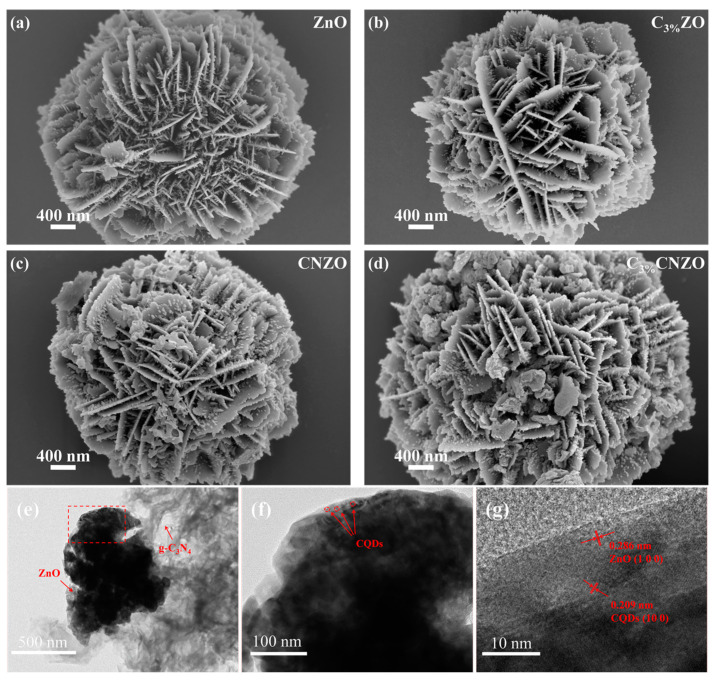
SEM images of (**a**) ZnO; (**b**) C_3%_ZO; (**c**) CNZO; and (**d**) C_3%_CNZO. TEM images of (**e**,**f**) C_3%_CNZO; and HRTEM image of (**g**) C_3%_CNZO.

Figure S1 shows the SEM image of g-C_3_N_4_, revealing that g-C_3_N_4_ exhibits an irregular lamellar structure. In Figure 1a, it can be observed that ZnO exhibits a lamellar flower-like structure, while there are tiny nano-cone structures on the surface of the lamellar structure. In Figure 1b, it can be observed that C_3%_ZO exhibits a lamellar flower-like structure with tiny nano-cone structures on the surface of the lamellar layers. This morphology and structure were similar to those of ZnO. Figure 1c depicts the SEM image of CNZO, where g-C_3_N_4_ was irregularly distributed on the surface of the flower-like ZnO material. Figure 1d shows the SEM image of C_3%_CNZO, where g-C_3_N_4_ was still irregularly distributed on the surface of the lamellar flower-like ZnO material. However, due to the small particle size of the CQDs, it cannot be observed in the SEM image. The EDS images of C_3%_CNZO are shown in Figure S2, from which the presence of Zn, O, C, and N elements can be observed. The Zn and O elements correspond to the flower-like ZnO, while the C and N elements originate from the g-C_3_N_4_ and CQDs. From the TEM images of C_3%_CNZO (Figure 1e,f), g-C_3_N_4_, ZnO, and CQDs in the photocatalyst can be observed. The HRTEM image of CQDs is shown in Figure S3, where it can be observed that the crystal plane spacing of CQDs was 0.209 nm, which corresponds to the (1 0 0) lattice plane of graphitic carbon [47]. The results show that the carbon cores of the CQDs had graphite-like sp^2^ hybridized carbon nano-structural domains, and the formation of the crystal structure were caused by the higher temperature and vapor pressure in the synthesis reaction [48]. In Figure S4b, it can be observed that the lattice spacing of 0.331 nm corresponds to the (0 0 2) crystallographic plane of g-C_3_N_4_ [49]. As shown in Figure 1g, the HRTEM image of C_3%_CNZO reveals the presence of CQDs. Two kinds of lattice fringes can be observed from the HRTEM image, in which the lattice spacing of 0.286 nm corresponds to the (1 0 0) crystallographic plane of ZnO, while the lattice spacing of 0.209 nm for the (1 0 0) crystallographic plane of CQDs. The successful introduction of CQDs can be demonstrated by the HRTEM test results.

**Figure 2:**
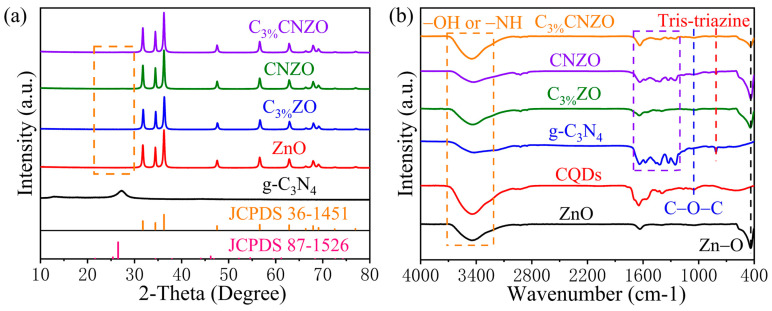
(**a**) XRD patterns of g-C_3_N_4_, ZnO, C_3%_ZO, CNZO, and C_3%_CNZO; (**b**) FTIR spectra of ZnO, CQDs, g-C_3_N_4_, C_3%_ZO, CNZO, and C_3%_CNZO.

The XRD spectra of the prepared samples are shown in Figure 2a, where the pure g-C_3_N_4_ exhibits two distinct diffraction peaks, at 2θ = 27.3° and 13.1°. The diffraction peaks at 27.3° were attributed to the interlayer stacking structure of aromatic segments (0 0 2). The diffraction peaks at 13.1° were due to the in-plane structural packing motif of tri-s-triazine units (1 0 0) (JCPDS 87-1526) [50]. For pure ZnO, all the diffraction peaks belong to the hexagonal wurtzite structure (JCPDS 36-1451) [51]. The peaks at 2θ = 31.8°, 34.5°, 36.3°, 47.6°, 56.6°, 62.9°, and 68.0° belong to the (1 0 0), (0 0 2), (1 0 1), (1 0 2), (1 1 0), (1 0 3), and (1 1 2) crystal planes, respectively. There were no impurity peaks in all these samples. However, due to the low content of g-C_3_N_4_ in the heterojunctions, it was difficult to observe the relevant diffraction peaks about g-C_3_N_4_. By magnifying the images in the range of 2θ = 22°–30°, as shown in Figure S5, it can be observed that the diffraction peaks at 2θ = 27.3° corresponds to the (0 0 2) lattice plane of g-C_3_N_4_. The FTIR spectra of ZnO, CQDs, g-C_3_N_4_, C_3%_ZO, CNZO, and C_3%_CNZO are shown in Figure 2b. The relatively broad band of 3000–3600 cm^−1^ was attributed to the stretching vibrations triggered by −NH groups or −OH groups in the material. The sharp absorption bands at 1200–1700 cm^−1^ and 808 cm^−1^ correspond to the stretching vibration of the heptazine heterocyclic ring (C_6_N_7_) units and the respiration vibration of the s-triazine in g-C_3_N_4_, respectively [52]. The peak at 437 cm^−1^ corresponds to the stretching vibration of the Zn−O bond in ZnO. As shown in Figure S6, the FTIR spectra of C_3%_ZO, CNZO, and C_3%_CNZO revealed that the peak at 1050 cm^−1^ corresponds to the C−O−C stretching vibration in CQDs. This indirectly indicates the successful incorporation of CQDs into the materials.

As shown in Figure S7, the N_2_ adsorption–desorption isotherms of g-C_3_N_4_, ZnO, C_3%_ZO, CNZO, and C_3%_CNZO exhibited type IV adsorption–desorption isothermal isotherms with H_3_ hysteresis loops. The specific surface area and porosity of the materials are provided in Table S1. Due to the low amounts of both g-C_3_N_4_ and CQDs, the specific surface areas and porosity of C_3%_ZO, CNZO, and C_3%_CNZO were similar to those of ZnO.

**Figure 3:**
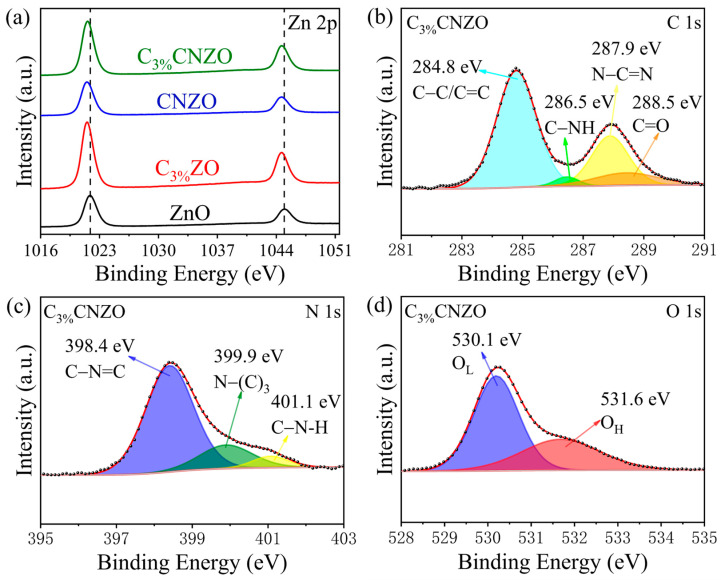
(**a**) High-resolution spectra of the Zn element; (**b**–**d**) high-resolution spectra of the C, N and O element in C_3%_CNZO, respectively.

In Figure S8a, Zn, O, C, and N elements can be observed from the XPS full spectrum of g-C_3_N_4_, ZnO, C_3%_ZO, CNZO, and C_3%_CNZO. Figure 3a shows the high-resolution spectra of the Zn element, which demonstrates a shift toward lower binding energies for the Zn 2p peak in C_3%_ZO, CNZO, and C_3%_CNZO compared to the ZnO binding energy. This suggests that electron transfer occurs between the CQDs, g-C_3_N_4_ and ZnO, indicating that the catalyst is not simply physical mixture. Figure 3b and Figure S8b show the high-resolution spectra of the C elements of C_3%_CNZO and g-C_3_N_4_, respectively. Compared with g-C_3_N_4_, a peak with a binding energy of 288.5 eV appeared in C_3%_CNZO, which corresponds to the C=O binding energy in CQDs and indirectly indicates the introduction of CQDs. Figure 3c shows the high-resolution spectra of the N elements of C_3%_CNZO with three distinct peaks observed at 398.4 eV, 399.9 eV, and 401.1 eV, corresponding to C−N=C, N−(C)_3_, and C−N−H, respectively. Figure 3d shows the high-resolution spectra of the O elemental of C_3%_CNZO, where the O 1s peaks at binding energies of 530.1 eV and 531.6 eV correspond to the lattice oxygen and surface adsorbed oxygen, respectively. Changes in the binding energies of the corresponding peaks in the high-resolution spectra of the C, N, and O elements were observed. It can be demonstrated that electron transfer occurs between CQDs, g-C_3_N_4_, and ZnO, rather than a simple physical mixture.

### 3.2. Photoelectric Chemical Performance Analysis

**Figure 4:**
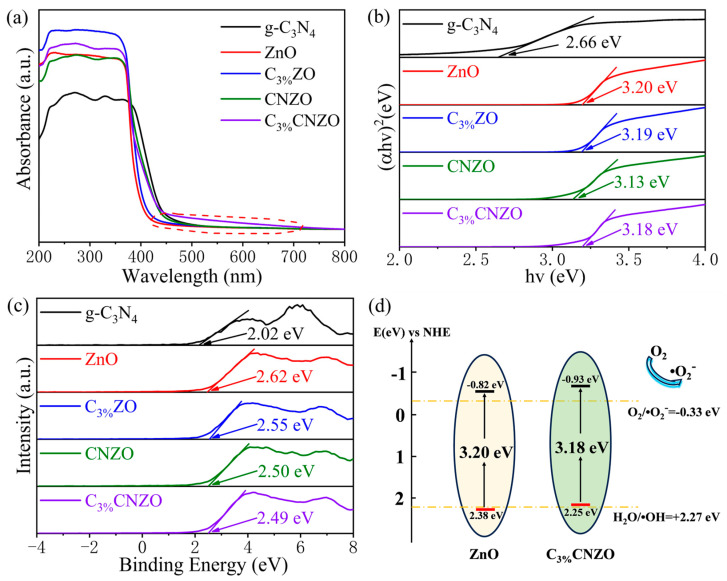
(**a**) UV-Vis absorption spectrum of g-C_3_N_4_, ZnO, C_3%_ZO, CNZO, and C_3%_CNZO; (**b**) Eg curves of g-C_3_N_4_, ZnO, C_3%_ZO, CNZO, and C_3%_CNZO; (**c**) E _(VB, XPS)_ spectra of g-C_3_N_4_, ZnO, C_3%_ZO, CNZO, and C_3%_CNZO; and (**d**) photocatalytic mechanism diagram of C_3%_CNZO.

Figure 4a shows the UV-Vis spectra of g-C_3_N_4_, ZnO, C_3%_ZO, CNZO, and C_3%_CNZO, from which the joining of g-C_3_N_4_ led to the red-shift of the absorption bands of ZnO. At the same time, the presence of CQDs generated a visible absorption band of the catalyst. The phenomenon indicates that the addition of g-C_3_N_4_ and CQDs can enhance the light utilization of ZnO, which is favorable for improving its photocatalytic performance. The band gap of the material can be calculated according to the following equation:

$${\left(\alpha h\nu \right)}^{2}=A(h\nu -E_{g})$$

where A is a constant, *h* and *ν* are Planck constant and electron frequency, respectively, and *E_g_* is the semiconductor forbidden bandwidth, which can be obtained from the material by linearly fitting *(αhv)^2^* to *hν*. In Figure 4b, the calculated Eg of g-C_3_N_4_, ZnO, C_3%_ZO, CNZO, and C_3%_CNZO are 2.66 eV, 3.20 eV, 3.19 eV, 3.13 eV, and 3.18 eV, respectively, from which it can be observed that the band gap of ZnO decreased due to the addition of CQDs and g-C_3_N_4_. However, the changes are not obvious due to the small amounts of CQDs and g-C_3_N_4_. Figure 4c shows the valence band diagrams of g-C_3_N_4_, ZnO, C_3%_ZO, CNZO, and C_3%_CNZO. The valence band data relating to the standard hydrogen electrode can be obtained according to the following equation:

$$E_{\left(\mathrm{VB},\mathrm{NHE}\right)}=E_{\left(\mathrm{VB},\mathrm{XPS}\right)}+\delta -4.44$$

where *E*_(*VB,NHE*)_ is the valence band potential at the standard hydrogen electrode, and *E*_(*VB,XPS*)_ is the valence band potential tested with XPS and δ is the instrument work function, which was 4.20 eV for the equipment used in this test. Based on the above formula, the valence band potentials of g-C_3_N_4_, ZnO, C_3%_ZO, CNZO, and C_3%_CNZO can be calculated to be 1.78 eV, 2.38 eV, 2.31 eV, 2.26 eV, and 2.25 eV, respectively, at the standard hydrogen electrode. The potential at the standard hydrogen electrode for H_2_O/•OH was 2.27 eV. The CNZO and C_3%_CNZO systems were unable to produce •OH radicals. The conduction band of the catalyst can be calculated from the following equation:

$$E_{\left(\mathrm{CB},\mathrm{NHE}\right)}=E_{\left(\mathrm{VB},\mathrm{NHE}\right)}-\mathrm{Eg}$$

where *E*_(*CB,NHE*)_ is the conduction band potential at the standard hydrogen electrode. The calculation of the above formula leads to the conduction bands of −0.88 eV, −0.82 eV, −0.88 eV, −0.87 eV, and −0.93 eV for g-C_3_N_4_, ZnO, C_3%_ZO, CNZO, and C_3%_CNZO, respectively. O_2_/•O_2_^−^ had a potential of −0.33 eV at the standard hydrogen electrode. The CNZO and C_3%_CNZO systems can produce •O_2_^−^ radicals. With the above calculations, the energy band structure of the heterojunction is shown in Figure 4d.

To better investigate the effective degradation mechanism of CIP, we characterized the photo-electrochemical properties of the samples using transient photocurrent and electrochemical impedance spectroscopy. In Figure S9a, C_3%_CNZO has the highest photocurrent density, indicating that C_3%_CNZO can produce more photogenerated electrons. As shown in the test results of Figure S9b, C_3%_CNZO exhibits the smallest radius of Nyquist curve, indicating that its interfacial charge transfer resistance is the smallest. The results of photocurrent density curves and electrochemical impedance spectra show that the CQDs-modified enhanced layered g-C_3_N_4_/flower-like ZnO can generate more photogenerated electron–hole pairs under the effect of light. The CQDs can be used as a kind of electron-transporting medium, which is more favorable for the transfer of electrons.

### 3.3. Photocatalytic Performance

**Figure 5:**
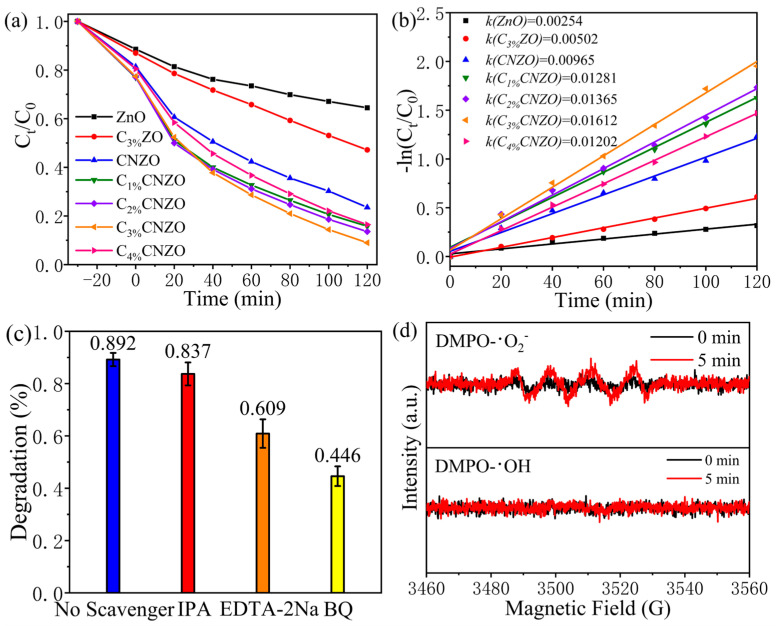
(**a**) Photocatalytic performance of ZnO, C_3%_ZO, CNZO, and C_x%_CNZO (x = 1, 2, 3, 4); (**b**) corresponding kinetic curves of the photodegradation rate; (**c**) free radical capture experiment; and (**d**) Spin-trapping EPR spectra of C_3%_CNZO under natural sunlight.

The photocatalytic performance of C_x%_CNZO (x = 1, 2, 3, 4) photocatalysts were evaluated using CIP as a contaminant. The adsorption performance of the catalysts is shown in Figure S10a, from which the catalysts can reach the adsorption–desorption equilibrium within 30 min. After 30 min in the dark, the photocatalysts were driven to degrade CIP at room temperature using a 300 W xenon lamp as a simulated light source. In Figure S10b, we can see that CIP gradually degraded by C_3%_CNZO over time. And in Figure 5a, it can be observed that ZnO, C_3%_ZO, CNZO, C_1%_CNZO, C_2%_CNZO, C_3%_CNZO, and C_4%_CNZO can remove 35.5%, 52.8%, 76.5%, 84.2%, 86.4%, 91.0%, and 83.4% of CIP within 120 min, respectively. The C_3%_CNZO had the best performance compared with pure ZnO. According to Figure 5b, it can be found that the kinetics of the catalytic degradation of CIP by C_x%_CNZO (x = 1, 2, 3, 4) conform to the first-order kinetic model. Among them, the kinetic constant of C_3%_CNZO reached the maximum, and its rate constant was 6.35 times more than that of ZnO, which shows that the performance of the heterojunction was greatly improved compared to that of pure ZnO. It was shown that the degradation effect increased and then decreased with the addition of CQDs. The phenomenon may be attributed to the fact that CQDs can act as electron acceptors. In the low content of CQDs, the electrons generated by g-C_3_N_4_ and ZnO can be transferred to the CQDs, which effectively separates the photogenerated electron–hole pairs (the result is consistent with a transient photocurrent) and improves the photocatalytic performance of the heterojunctions. However, excessive CQDs can also become the complex center of photogenerated electron–hole pairs. Thus, making the photocatalysts exhibited a decrease in photocatalytic performance. We summarized the catalytic performance of various photocatalysts for CIP and compared it with our work in Table S3.

From Figure 5c, the degradation performance of C_3%_CNZO remained largely unaffected when IPA was added, indicating that •OH radicals were not the main active species in this catalytic system. There was a significant decrease in the degradation rate with the addition of EDTA-2Na, indicating that h^+^ plays a role in this system. The degradation rate decreased most significantly after capturing •O_2_^−^ radical with BQ, demonstrating that the •O_2_^−^ radical is the main active species in this system. In Figure S11, when Ar was blown into the catalytic process, the catalytic performance decreased significantly. All results show that the •O_2_^−^ radical is the main active species in this system.

**Figure 6:**
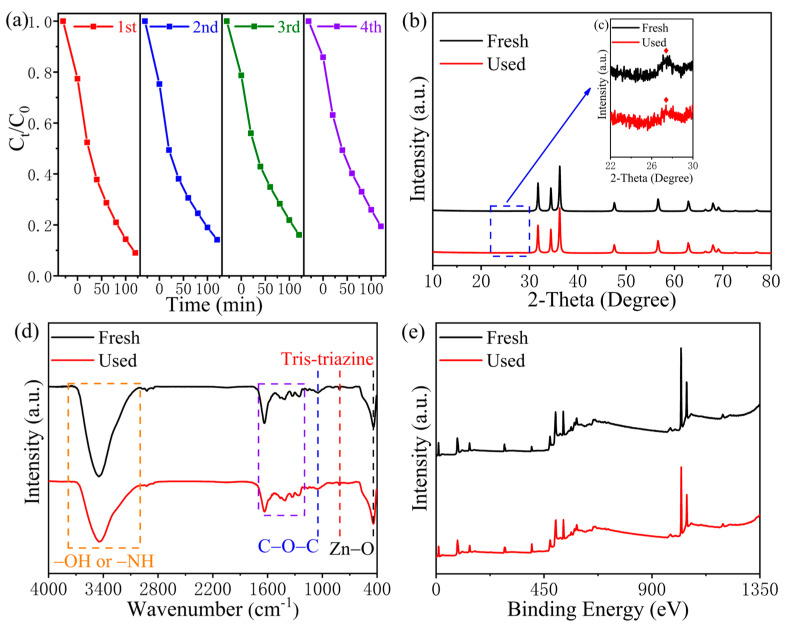
(**a**) Cycling performance of C_3%_CNZO; (**b**,**c**) XRD patterns of C_3%_CNZO after four cycles; (**d**) FTIR spectra of C_3%_CNZO after four cycles; and (**e**) XPS full spectrum of C_3%_CNZO after four cycles.

As shown in Figure 5d, to explicitly explore the generation of active species in C_3%_CNZO photocatalysts during photodegradation, it can be observed by EPR active species capture experiments. In these experiments, DMPO was used as a trapping agent for •O_2_^−^ radicals and •OH radicals to investigate the generation of active species in the C_3%_CNZO photocatalyst. In Figure 6a, the DMPO-•O_2_^−^ adduct exhibits strong characteristic signal peaks with a 1:1:1:1 intensity ratio, indicating the presence of •O_2_^−^ radicals. The DMPO-•OH adduct did not exhibit a 1:2:2:1 intensity ratio signal, indicating that no •OH radicals were generated in the system. The above results indicate that a significant number of •O_2_^−^ radicals were effectively produced in the C_3%_CNZO system, which is consistent with both the band gap calculations and the active species trapping experiments.

As shown in Figure 6a, the cycling performance test of C_3%_CNZO demonstrates that the removal rate of C_3%_CNZO remains above 80% after four cycles. The phenomenon indicates that the catalyst displays excellent cycling stability. The degradation in cycling performance is hypothesized to result from the shedding of some CQDs during the process. Figure S12 shows the SEM images of C_3%_CNZO after four cycles, from which it can be observed that ZnO still maintains a lamellar flower-like structure, and the nano-conical structure still remains on the surface of the lamellar layers. Additionally, the g-C_3_N_4_ was irregularly distributed on the surface of the lamellar flower-like ZnO material, indicating that the microscopic morphology of C_3%_CNZO was not damaged. As shown in Figure 6b,c, the diffraction peaks of C_3%_CNZO were consistent with those of the sample before used, indicating that the crystal structure of C_3%_CNZO remained intact after four cycles. As shown in Figure 6d, the FTIR spectrum of C_3%_CNZO after four cycles revealed no significant changes in the functional group structure, demonstrating that the surface functional groups of C_3%_CNZO remained largely unaffected. In Figure 6e, the XPS full spectrum of C_3%_CNZO after four cycles illustrates that the chemical states of the relevant elements remained largely unchanged. The heterojunction demonstrates good structural stability, as evidenced by the analysis of its cycling performance, SEM, XRD, FTIR, and XPS.

### 3.4. Ciprofloxacin Degradation Pathway

**Scheme 2:**
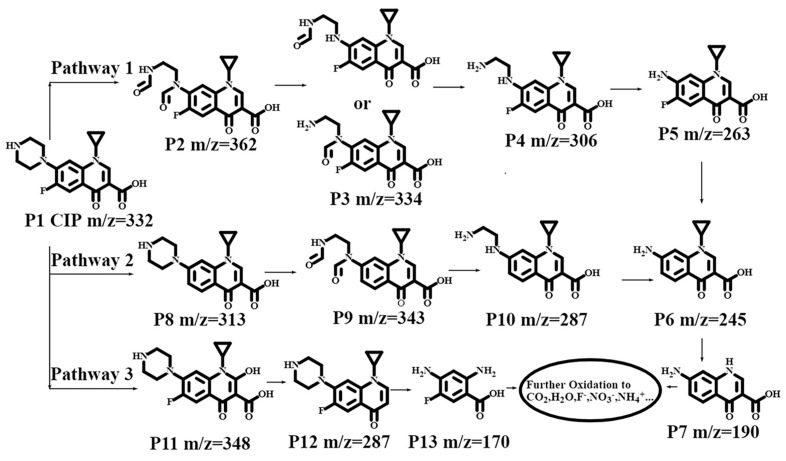
Possible degradation pathways of CIP during C_3%_CNZO catalysis.

To further investigate the degradation process of CIP, the main intermediates were identified using the LC-MS technique. The mass spectra of the possible molecular intermediates at reaction times of 0 min, 60 min, and 120 min are shown in Figures S13 and S14. Meanwhile, the molecular and structural formulas of the main chemical components identified in the proposed mass spectra are listed in Table S2. Based on the data analysis and the related literature [53,54], a reasonable degradation pathway for CIP was proposed, as shown in Scheme 2. It is hypothesized that there are three possible pathways for the decomposition of CIP. Pathway 1: P1 (CIP *m*/*z* = 332) → P2 (*m*/*z* = 362) → P3 (*m*/*z* = 334) → P4 (*m*/*z* = 306) → P5 (*m*/*z* = 263) → P6 (*m*/*z* = 245) → P7 (*m*/*z* = 190). In this degradation process, there was a net loss of −C_2_H_2_ at the piperazinyl substituent of CIP, which obtained the desethylene ciprofloxacin (P4). The N (1) and N (4) atoms in the piperazine ring could be easily attacked by h^+^, leading to the initial opening of the piperazine ring, which formed the dialdehyde derivative P2. This was followed by the loss of one formaldehyde molecule to form the keto-derivatives P3 and P3. Then, the other formaldehyde was eliminated leading to the formation of P4. Next, aniline P5 was generated, resulting in the complete destruction of the piperazinyl ring of CIP. Finally, defluorination and decyclopropane oxidized P5 to P6 and P7. Pathway 2: P1 (CIP *m*/*z* = 332) → P8 (*m*/*z* = 313) → P9 (*m*/*z* =343) → P10 (*m*/*z* = 287) → P6 (*m*/*z* = 245) → P7 (*m*/*z* = 190). P8 (*m*/*z* = 313) is a defluorination product, which was formed by the breakage of the C-F bond. Then, the piperazine underwent ring opening, resulting in a degradation process like pathway 1. Pathway 3: P1 (CIP *m*/*z* = 332) → P11 (*m*/*z* = 348) → P12 (*m*/*z* = 287) → P13 (*m*/*z* = 170). The pathway primarily involves the removal of the carboxyl group and the cleavage of the quinolone ring. However, the intermediates subsequently produced were too abundant to be clearly identified. Nevertheless, a possible pathway was summarized by our analysis. First, CIP was attacked by hydroxyl radicals, resulting in the formation of the hydroxylated intermediate P11. This intermediate underwent decarboxylation to form P12. Eventually, the quinolone ring was damaged, leading to the formation of the ring-opening product P13. Finally, all by-products could undergo further degradation into small organic molecules, ultimately resulting in the formation of carbon dioxide and water.

### 3.5. Photocatalytic Mechanism

**Figure 7:**
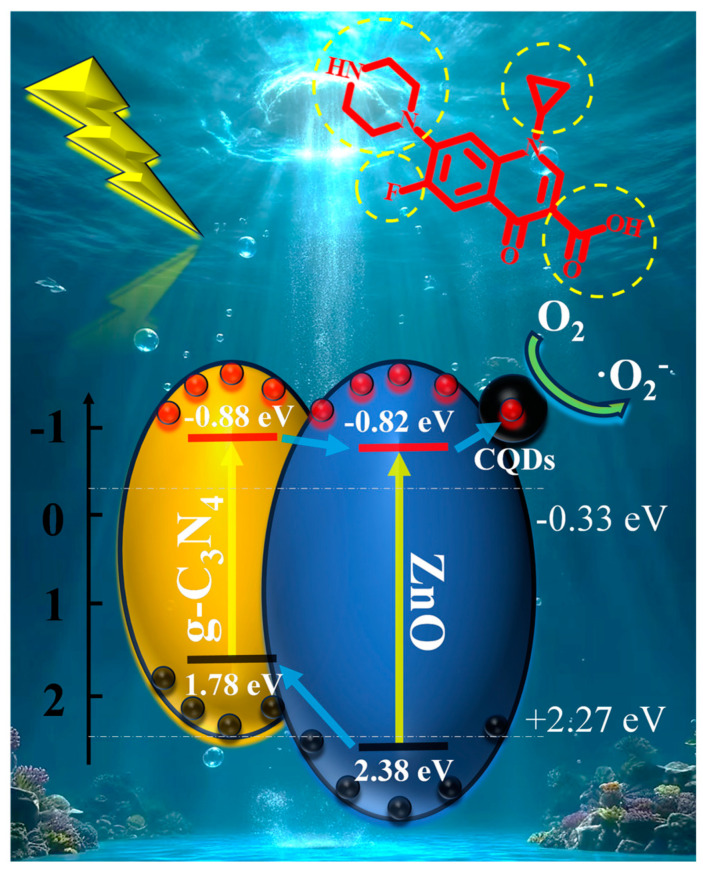
Electron transfer mechanism of C_3%_CNZO.

According to the experimental results above, C_3%_CNZO demonstrates excellent photocatalytic degradation performance and good cyclic degradation stability for CIP. Through active species trapping and EPR characterization, an electron transfer mechanism for C_3%_CNZO was proposed, as shown in Figure 7. The electron transfer path of CQDs-modified layered g-C_3_N_4_/flower-like ZnO is consistent with a type II heterojunction. The photocatalytic mechanism mainly includes three steps. (1) Under the excitation of sunlight, the electrons (e^−^) located in the valence band (VB) of g-C_3_N_4_ and ZnO absorb sufficient photon energy to migrate into the conduction band (CB), leaving behind the hole (h^+^) in VB. The photogenerated holes can oxidize CIP molecules directly. (2) Un-reorganized photogenerated electron–hole pairs will migrate to the surface of the material and the electrons located in the conduction band of g-C_3_N_4_ will transfer to the conduction band of ZnO. Moreover, the presence of CQDs, which can act as an electronic medium, facilitates the migration of electrons from the conduction band of ZnO to the CQDs, effectively preventing the recombination of electron–hole pairs [55,56,57]. The electrons on the CQDs will react with oxygen absorbed on the semiconductor, forming superoxide anion radical (•O_2_^−^). Then, the •O_2_^−^ will react with water to subsequently form hydroxyl radical (•OH). Meanwhile, the holes located in the valence band of ZnO migrate into the valence band of g-C_3_N_4_. However, the oxidation potential of h^+^ is lower than that required to oxidize water to hydroxyl radicals (•OH). The hydroxyl radicals (•OH) cannot directly produce in this system. (3) These reactive radicals further react with CIP molecules and mineralize them into nontoxic by-products, even CO_2_ and H_2_O. The relevant reactions that may be involved in the solution are listed below:

$$\mathrm{Catalyzer}+h\nu \to h^{+}+e^{-}\;\mathrm{Pollutant}+h^{+}\to {\left(\mathrm{Pollutant}\right)}^{+}\;e^{-}+O_{2}\to {•O}_{2}^{-}$$

## 4. Conclusions

In summary, CQDs-modified layered g-C_3_N_4_/flower-like ZnO (C_3%_CNZO) heterojunction photocatalysts were successfully prepared at room temperature. Under a 300 W xenon lamp, its CIP removal rate reached 91.0%, and its photocatalytic efficiency was significantly enhanced and demonstrated superior catalytic performance compared to pure ZnO. The photodegradation reaction data were consistent with the first-order kinetic model. This phenomenon can be attributed to the enhanced absorption of the visible part in the catalyst by g-C_3_N_4_. Meanwhile, as an electronic medium, CQDs can effectively separate the electron–hole pairs, thereby improving the photocatalytic performance. In addition, the prepared heterojunction exhibited excellent reusability and photostability. Regarding the removal rate of CIP, the removal rate of the photocatalyst remained above 80% even after four cycles. Based on the active species trapping and EPR tests, it was inferred that superoxide (•O_2_^−^) radicals were involved in the degradation process. Through the LC-MS analysis, three possible degradation pathways of CIP during the degradation process were identified and summarized. The above results indicate that this material has good potential for solar degradation of antibiotics and provides a new idea for the treatment of pollutants in water with ZnO substrate materials.

## Data Availability

Data are contained within the article and the Supplementary Materials.

## Supplementary Materials

The following supporting information can be downloaded at https://www.mdpi.com/article/10.3390/nano15070550/s1. The test method of LC-MS. Figure S1: SEM image of g-C_3_N_4_. Figure S2: The EDS mapping images of the C_3%_CNZO. Figure S3: The HRTEM image of CQDs. Figure S4: (a) The TEM image of C_3%_CNZO; (b) The HRTEM image of C_3%_CNZO. Figure S5: XRD image of the sample at diffraction angles of 22°–30°. Figure S6: Localized magnified view of FTIR. Figure S7: N_2_ adsorption–desorption isothermal curves of g-C_3_N_4_, ZnO, C_3%_ZO, CNZO, and C_3%_CNZO. Figure S8: (a) XPS full spectrum of g-C_3_N_4_, ZnO, C_3%_ZO, CNZO, and C_3%_CNZO; (b) high-resolution spectra of the C element in g-C_3_N_4_; (c) high-resolution spectra of the N element in g-C_3_N_4_; and (d) high-resolution spectra of the N element in ZnO. Figure S9: (a) Photocurrent density curves of ZnO, C_3%_ZO, CNZO, and C_3%_CNZO; (b) electrochemical impedance spectroscopy of ZnO, C_3%_ZO, CNZO, and C_3%_CNZO. Figure S10: (a) Absorption performance of ZnO, C3%ZO, CNZO, C3%CNZO; (b) UV-Vis absorption spectra of CIP at different times. Figure S11: The photocatalytic performance with Ar blowing. Figure S12: SEM images after four photocatalytic cycles of C_3%_CNZO. Figure S13: MS spectra of the CIP at different illumination times of 0 min, 60 min and 120 min. Figure S14: MS spectra of the possible intermediates during the degradation of CIP. Table S1: Specific surface area and porosity of g-C_3_N_4_, ZnO, C_3%_ZO, CNZO, C_3%_CNZO. Table S2: Chemical intermediates formed during CIP degradation. Table S3: The comparison of some recent literature reports on the photocatalytic degradation of CIP by various photocatalysts with our study. Refs. [58,59,60,61,62,63,64,65] are cited in the Supplementary Materials.
