# Supplementary material for: Enhanced Photocatalytic Activity of CQDs-Modified Layered g-C3N4/Flower-like ZnO Heterojunction for Efficient Degradation of Ciprofloxacin
Source: Nanomaterials (Basel). 2025 Apr 4;15(7):550. doi: 10.3390/nano15070550 (PMC11990431; doi:10.3390/nano15070550)
Supplement: Supplementary file 1 [file nanomaterials-15-00550-s001.zip › nanomaterials-3564165-supplementary.pdf]

Supplementary Materials

Enhanced photocatalytic activity of CQDs-modified Layered g-C_3N_4/ Flower-like ZnO heterojunction for efficient degradation of ciprofloxacin

Qing Liu [1], Wei Deng [1], Hai Zhang [1], Jiajun Fang [1], Yushi Xie [1], Congwen Liu [1], Xiaochen Han [1], Xiaoling Xu [1,2,]⋆ and Zuowan Zhou [1,2]

[1] School of Chemistry, Key Laboratory of Advanced Technologies of Materials (Ministry of Education), Southwest Jiaotong University, Chengdu, 610031, China

[2] Yibin Research institute, Southwest Jiaotong University, Yibin 644000, China

⋆ Correspondence: bihan_2001@163.com; Tel.: +86-13880418330

Test methods of LC-MS:

Degradation intermediates of CIP were analyzed using an ultra-high performance liquid chromatograph (UHPLC) (Aglient 1260 Infinity II Prime-6530 Q-TOF, USA) equipped with an Infinity Lab Poroshell 120 EC-C18 (3.0 × 100 mm) column and a Thermo Scientific Q Exactive. The injection volume was 10 μl and the flow rate was 0.5 ml/min. Mobile phase A: 0.1% formic acid in water and mobile phase B: acetonitrile. 0−1 min, 5% acetonitrile; 1−10 min, 5−40% acetonitrile; 10−20 min, 95% acetonitrile. The column temperature was 30 °C. The analytes were ionized by a heated electrospray (Dual-AJS) probe in positive ion mode. The mass spectral parameters were as follows $T_{Capillary}$ = 320 °C; $T_{Auxiliary\ gas}$ = 300 °C; spray voltage = 3.8 kV, and mass spectral scanning range m/z = 50−400.

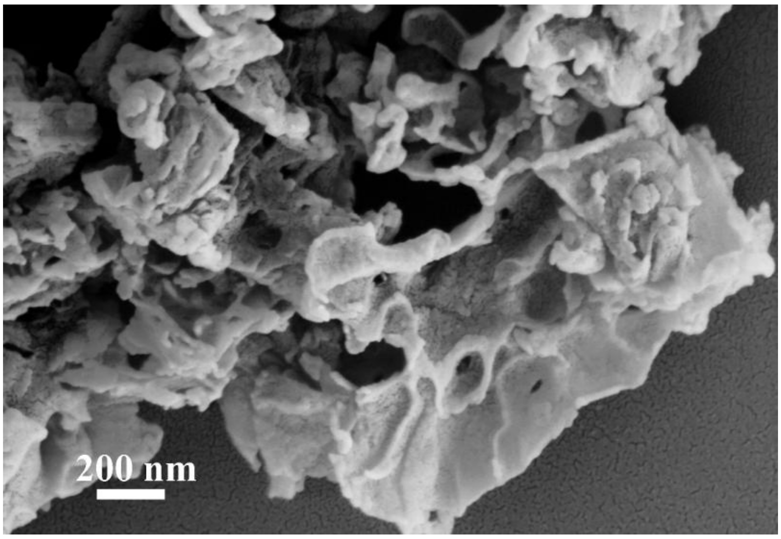

Figure S1. SEM image of g-C_3N_4.

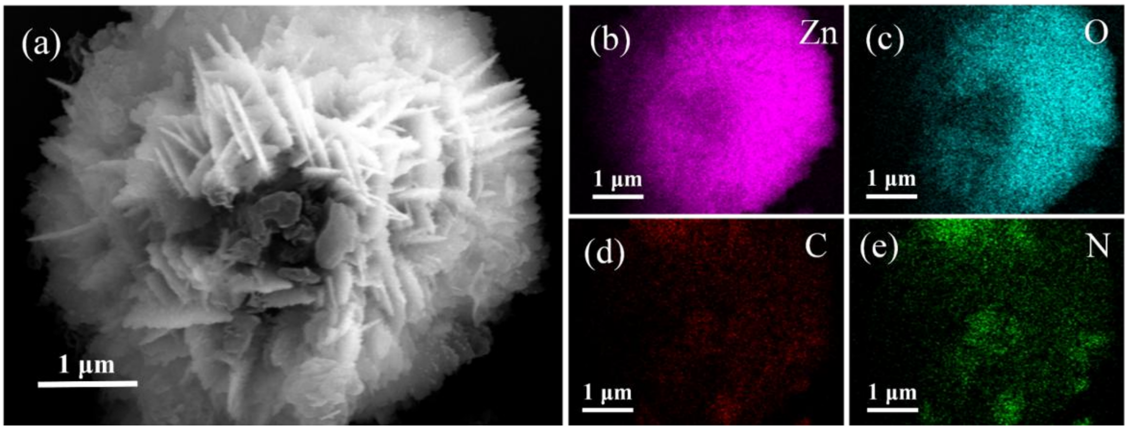

Figure S2. The EDS mapping images of the $C_{3\%}CNZO$.

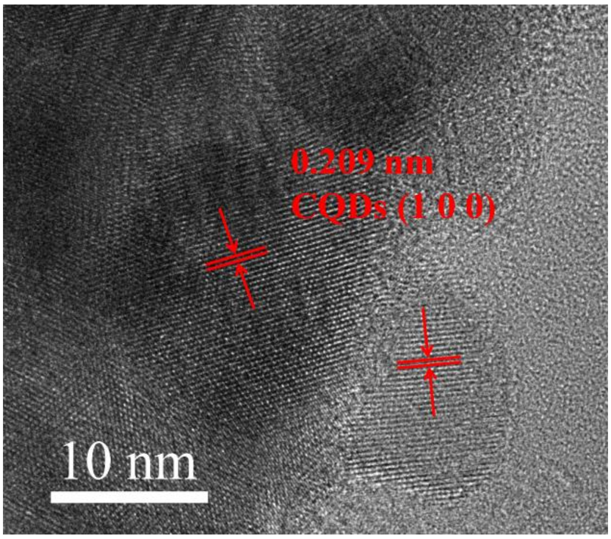

Figure S3. The HRTEM image of CQDs.

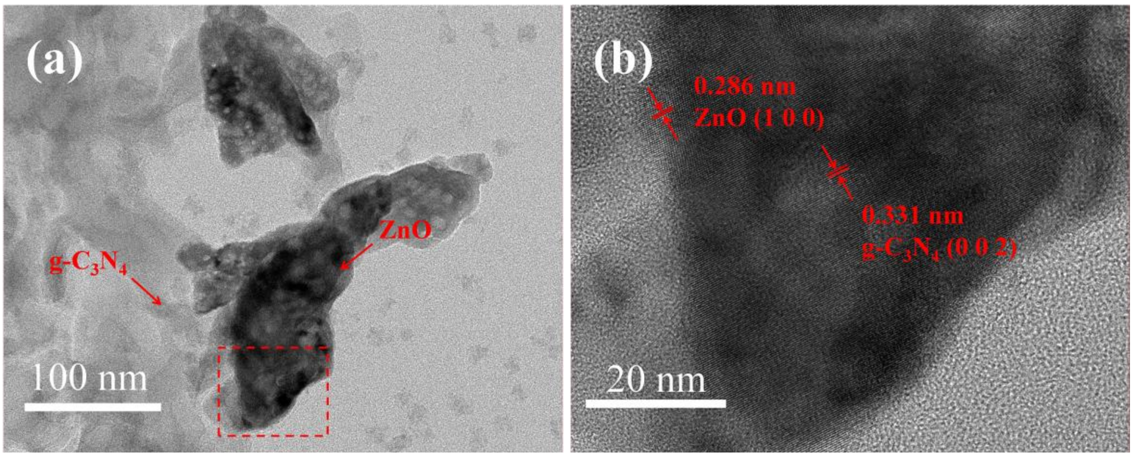

Figure S4. (a) The TEM image of $C_{3\%}CNZO$; (b) The HRTEM image of $C_{3\%}CNZO$.

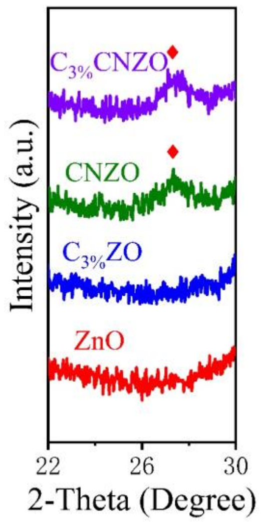

Figure S5. XRD image of the sample at diffraction angles of 22°−30°.

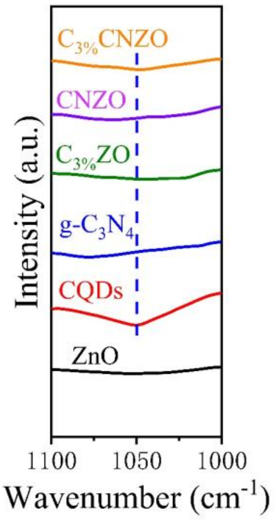

Figure S6. Localized magnified view of FTIR.

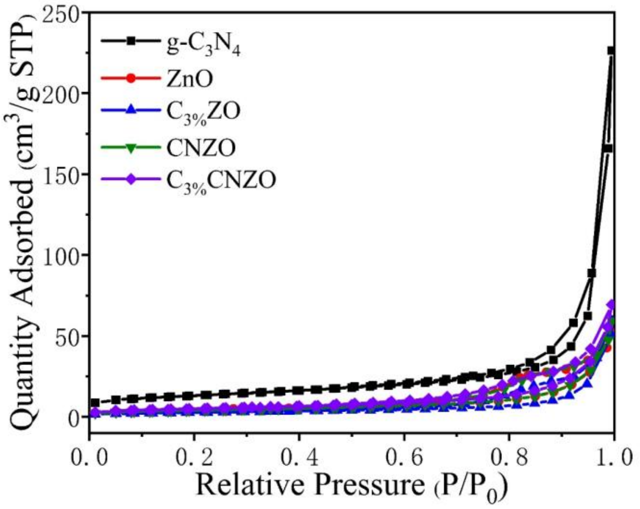

Figure S7. N_2 adsorption−desorption isothermal curves of g-C_3N_4, ZnO, $C_{3\%}$ZO, CNZO, $C_{3\%}$CNZO.

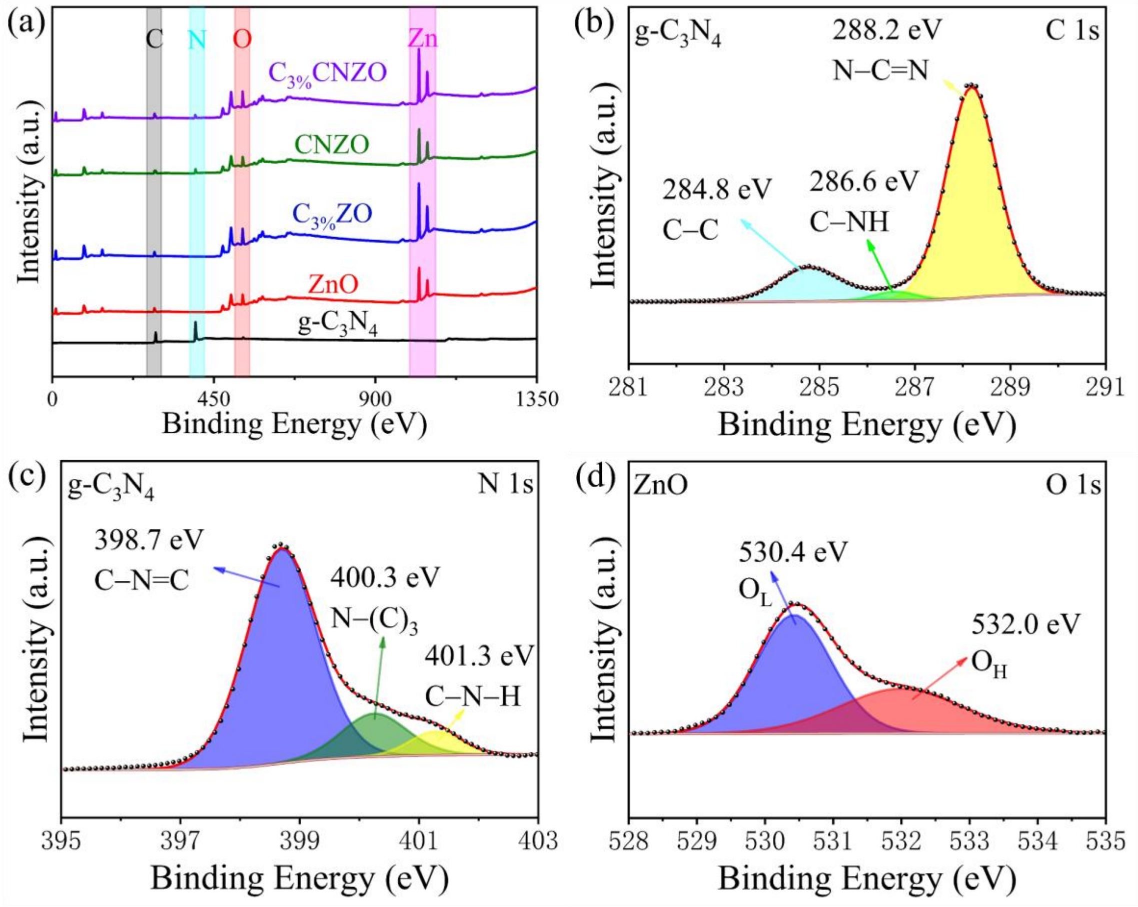

Figure S8. (a) XPS full spectrum of g-C_3N_4, ZnO, $C_{3\%}ZO$, CNZO, $C_{3\%}CNZO$; (b) high-resolution spectra of the C element in g-C_3N_4; (c) high-resolution spectra of the N element in g-C_3N_4; (d) high-resolution spectra of the N element in ZnO.

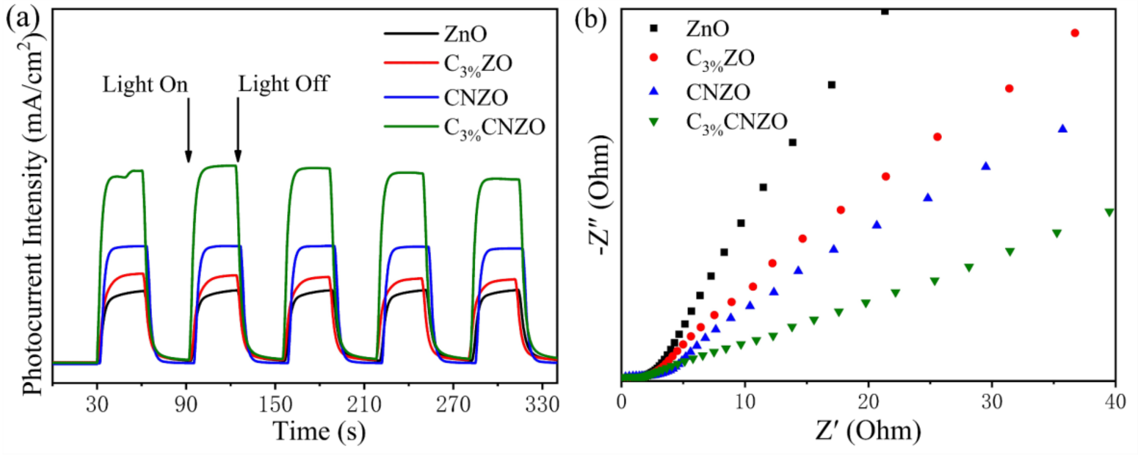

Figure S9. (a) Photocurrent density curves of ZnO, $C_{3\%}ZO$, CNZO, $C_{3\%}CNZO$; (b) electrochemical impedance spectroscopy of ZnO, $C_{3\%}ZO$, CNZO, $C_{3\%}CNZO$.

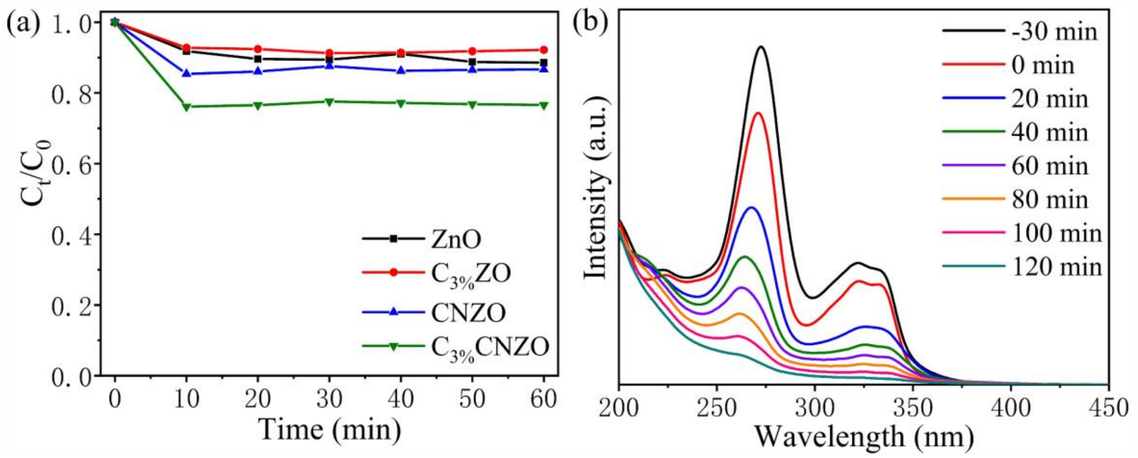

Figure S10. (a) Absorption performance of ZnO, $C_{3\%}ZO$, CNZO, $C_{3\%}CNZO$; (b) UV-Vis absorption spectra of CIP at different times.

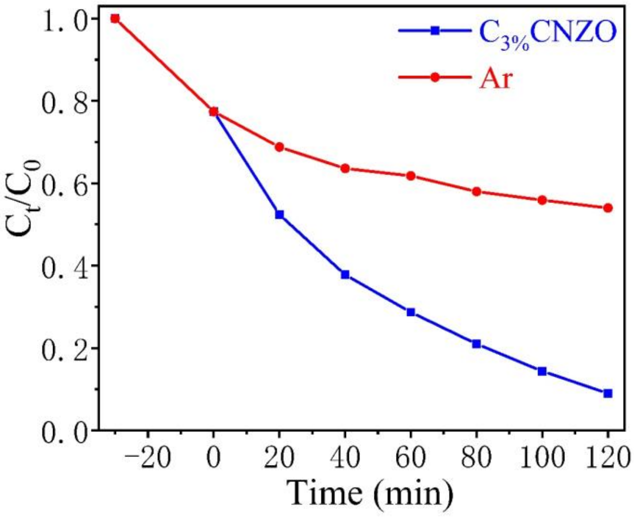

Figure S11. The photocatalytic performance with Ar blowing.

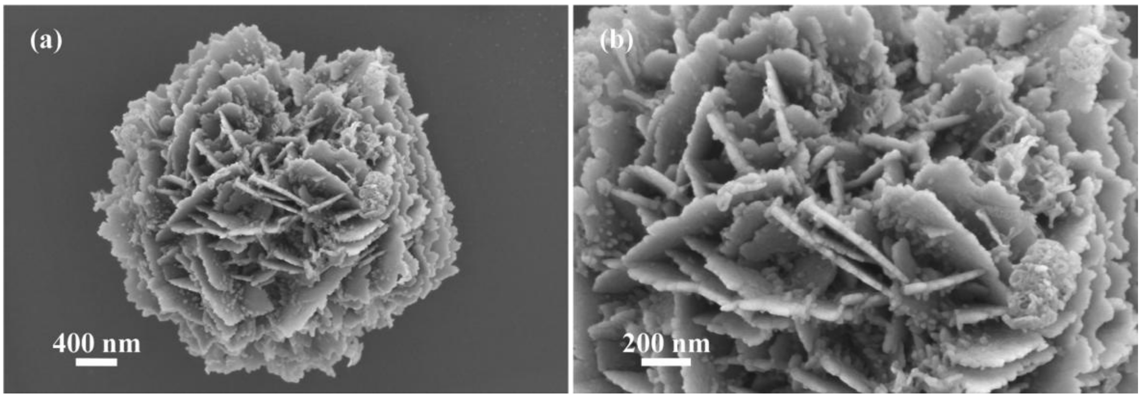

Figure S12. SEM images after four photocatalytic cycles of $C_{3\%}CNZO$.

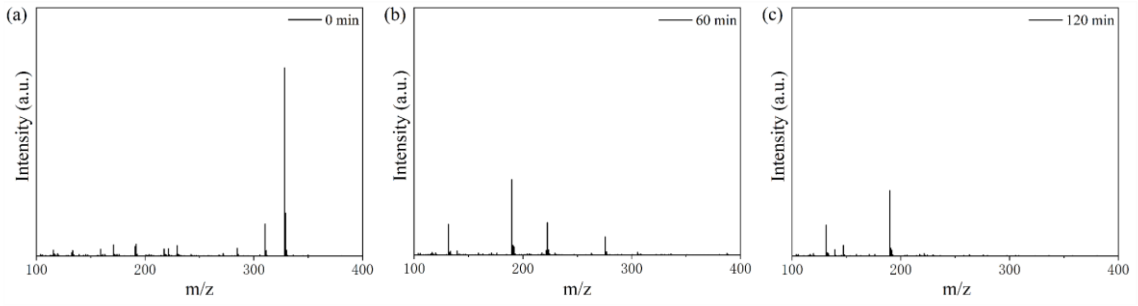

Figure S13. MS spectra of the CIP at different illumination times of 0 min, 60 min and 120 min.

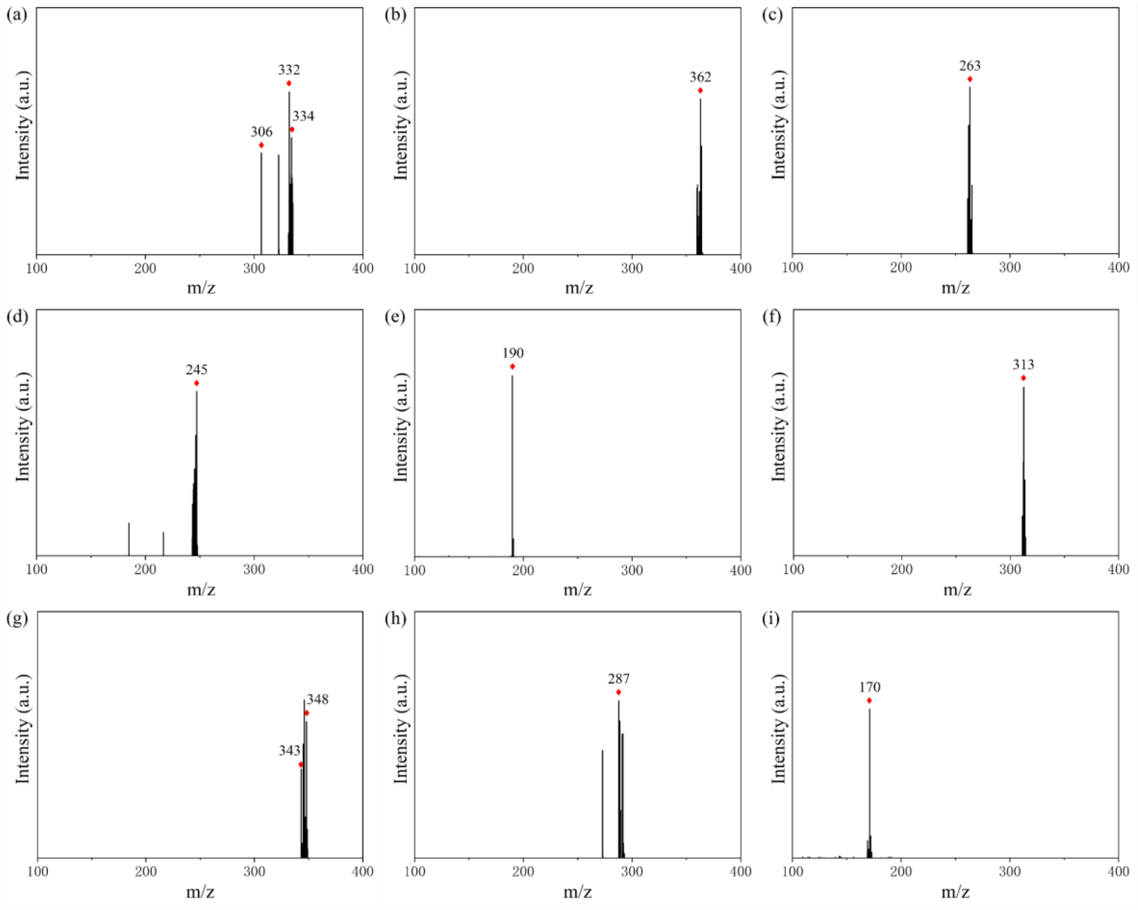

Figure S14. MS spectra of the possible intermediates during the degradation of CIP.

Table S1. Specific surface area and porosity of g-C_3N_4, ZnO, $C_{3\%}$ZO, CNZO, $C_{3\%}$CNZO.

	g-C_3N_4	ZnO	$C_{3\%}$ZO	CNZO	$C_{3\%}$CNZO
Specific surface area (m^2/g)	46.93	12.34	10.09	15.31	17.95
Porosity (m^3/g)	0.346	0.080	0.078	0.092	0.108

Table S2. Chemical intermediates formed during CIP degradation.

NO.	Molecular formula	m/z	Structural formula
P1	$C_{17}H_{18}N_3O_3$	332	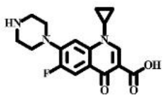
P2	$C_{17}H_{16}FN_3O_5$	362	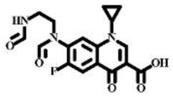
P3	$C_{16}H_{16}FN_3O_4$	334	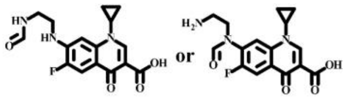
P4	$C_{15}H_{16}FN_3O_3$	306	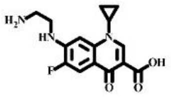
P5	$C_{13}H_{11}FN_2O_3$	263	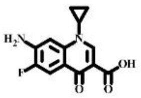
P6	$C_{13}H_{12}N_2O_3$	245	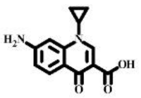
P7	$C_{10}H_7NO_3$	190	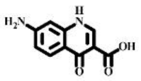
P8	$C_{17}H_{19}N_3O_3$	313	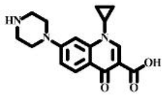
P9	$C_{17}H_{17}N_3O_5$	343	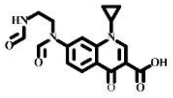
P10	$C_{15}H_{17}N_3O_3$	287	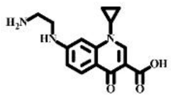
P11	$C_{17}H_{18}FN_3O_4$	348	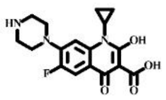
P12	$C_{16}H_{18}FN_3O$	287	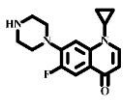
P13	$C_7H_7FN_2O_2$	170	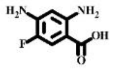

Table S3. The comparison of some recent literature reports on the photocatalytic degradation of CIP by various photocatalysts with our study.

Photocatalysis	Light source	The concentration of CIP (mg/L)	The concentration of Catalysis (g/L)	Degradation efficiency	Ref.
$Bi_2Ti_2O_7/TiO_2$/RGO	500 W Xenon lamp	10	0.25	95.0% in 180 min	[1]
CeO_2-Ag/AgBr	300 W Xenon lamp	10	1	93.05% in 120 min	[2]
Bi_2MoO_6/g-$C_3N_4/BiFeO_3$	500 W Xenon lamp with a 400 nm filter	12	1	95.0% in 180 min	[3]
Ti_3C_2 MXene Reinforced g-C_3N_4	300 W Xenon lamp with a 400 nm filter	10	2	93.3% in 150 min	[4]
Vo-Bi_2WO_6	300 W Xenon lamp with a 400 nm filter	20	1	90.0% in 360 min	[5]
Fe/BiOCl/RGO	500 W Xenon lamp with a 420 nm filter	10	0.25	86.0% in 180 min	[6]
Magnetic fly-ash	500 W tungsten lamp	10	10	74.0% in 120 min	[7]
TiO_2/g-C_3N_4	500 W Xenon lamp	3.3	0.2	91.0% in 120 min	[8]
CQDs/g-C_3N_4/ZnO	300 W Xenon lamp	10	1	91.0% in 120 min	This Work

Reference:

1. Li W, Zuo Y, Jiang L, Yao D, Chen Z, He G, et al. $Bi_2Ti_2O_7/TiO_2$/RGO composite for the simulated sunlight-driven photocatalytic degradation of ciprofloxacin. Materials Chemistry and Physics. 2020; 256. [CrossRef]
2. Wen X-J, Niu C-G, Zhang L, Liang C, Guo H, Zeng G-M. Photocatalytic degradation of ciprofloxacin by a novel Z-scheme CeO_2 − Ag/AgBr photocatalyst: Influencing factors, possible degradation pathways, and mechanism insight. Journal of Catalysis. 2018; 358: 141-54. [CrossRef]
3. Zhou Y, Zhang J, Wu D. Enhanced photocatalytic degradation of ciprofloxacin over Bi_2MoO_6/g-$C_3N_4/BiFeO_3$ heterojunction photocatalyst under visible light irradiation. Materials Science in Semiconductor Processing. 2022; 151. [CrossRef]
4. Zhang H, Xu J, Yuan Y, Guo Y, Tan X, Wang H, et al. Highly conductive Ti_3C_2 MXene reinforced g-C_3N_4 heterojunction photocatalytic for the degradation of ciprofloxacin: Mechanism insight. Separation and

Purification Technology. 2024; 330. [CrossRef]
5. Bai J, Zhang B, Xiong T, Jiang D, Ren X, Lu P, et al. Enhanced visible light driven photocatalytic performance of Bi_2WO_6 nano-catalysts by introducing oxygen vacancy. Journal of Alloys and Compounds. 2021; 887. [CrossRef]
6. Yin Y, Yao Y, Qian X, Sun M, Huang B, He G, et al. Fabrication of Fe/BiOCl/RGO with enhanced photocatalytic degradation of ciprofloxacin under visible light irradiation. Materials Science in Semiconductor Processing. 2022; 140. [CrossRef]
7. Lu Z, Zhu Z, Wang D, Ma Z, Shi W, Yan Y, et al. Specific oriented recognition of a new stable ICTX@Mfa with retrievability for selective photocatalytic degrading of ciprofloxacin. Catalysis Science & Technology. 2016; 6: 1367-77. [CrossRef]
8. Hu K, Li R, Ye C, Wang A, Wei W, Hu D, et al. Facile synthesis of Z-scheme composite of TiO_2 nanorod/g-C_3N_4 nanosheet efficient for photocatalytic degradation of ciprofloxacin. Journal of Cleaner Production. 2020; 253. [CrossRef]
